# A smart secured framework for detecting and averting online recruitment fraud using ensemble machine learning techniques

**DOI:** 10.7717/peerj-cs.1234

**Published:** 2023-02-08

**Authors:** Zahid Ullah, Mona Jamjoom

**Affiliations:** 1 Department of Information Systems, Faculty of Computing and Information Technology, King Abdulaziz University, Jeddah, Saudi Arabia; 2 Department of Computer Sciences, College of Computer and Information Sciences, Princess Nourah bint Abdulrahman University, Riyadh, Saudi Arabia

**Keywords:** Smart secured framework, Fraud detection, Online recruitment fraud, Ensemble ML methods, Prediction models

## Abstract

With the rise of the Internet and social media, information has become available at our fingertips. However, on the dark side, these advancements have opened doors for fraudsters. Online recruitment fraud (ORF) is one of the problems created by these modern technologies, as hundreds of thousands of applicants are victimized every year globally. Fraudsters advertise bogus jobs on online platforms and target job hunters with fake offerings such as huge salaries and desirable geographical locations. The objective of these fraudsters is to collect personal information to be misused in the future, leading to the loss of applicants’ privacy. To prevent such situations, there is a need for an automatic detecting system that can distinguish between real and fake job advertisements and preserve the applicants’ privacy. This study attempts to build a smart secured framework for detecting and preventing ORF using ensemble machine learning (ML) techniques. In this regard, four ensemble methods—AdaBoost (AB), Xtreme Gradient Boost (XGB), Voting, and Random Forest (RF)—are used to build a detection framework. The dataset used was pre-processed using several methods for cleaning and denoising in order to achieve better outcomes. The performance evaluation measures of the applied methods were accuracy, precision, sensitivity, F-measure, and ROC curves. According to these measures, AB performed best, followed by XGB, voting, and RF. In the proposed framework, AB achieved a high accuracy of 98.374%, showing its reliability for detecting and preventing ORF. The results of AB were compared to existing methods in the literature validating the reliability of the model to be significantly used for detecting ORF.

## Introduction

The rise of the Internet and social media have increased the likelihood of online recruitment and facilitated several organizations to use automated intelligent systems for recruiting new candidates, as this is a robust, accurate, and cost-efficient process (Vidros et al., 2017). The systems, servers, and clouds they utilize are managed by recruitment managers. However, the rapid increase in online job advertisements has maximized the number of fraudulent job postings, leading job hunters to experience harassment (Habiba, Islam & Tasnim, 2021). Therefore, the exposure of this kind of information on an online platform leads to another form of catastrophe that may result in a potential loss of privacy for candidates as well as harm to the companies’ reputations (Vidros et al., 2017). Moreover, several other risk factors are involved in this broad concern, such as scams, fraud, and the adoption of such systems (Alghamdi & Alharby, 2019). Around $4 trillion is spent on cybercrimes every year, and with the emergence of new violations, there is a strong need for records protection to impede abuse and uphold authenticity and accessibility (AsmithaShree et al., 2021).

A recruitment scam involves the deceitful intention of an individual or group who targets job hunters by posting bogus job advertisements to achieve malevolent objectives (Mehboob & Malik, 2021). These scams are undertaken in a deceitful manner, such as by showing attractive salaries to the applicants and collecting their personal information, asking for online testing and then taking them to a fraudulent site where their bank information is collected, and collecting survey system history by sharing viruses and malware to the applicants’ computers (Mehboob & Malik, 2021). The latest survey in the UK shows that more than 67% of applicants who search for jobs online lack awareness of job scams and are at high risk of being defrauded by them. Around 700,000 job hunters were victims of job scams, losing a combined total of more than $50,000 (Habiba, Islam & Tasnim, 2021). The Federal Trade Commission (Terrell, 2021) registered more than 100,000 complaints of fraud from job hunters between 2014 and 2019 (Goyal, Sachdeva & Kumaraguru, 2021). The organizations trap the young talent to defraud them of their money and personal information (Ranparia, Kumari & Sahani, 2020). In this way, cybercriminals collect applicants’ information to resell or use later for their purposes (Anita et al., 2021).

As discussed above, employment scams and fraudulent job postings are common, and fraudsters have a variety of reasons for collecting and misusing applicants’ information. Such crimes occur across social media and other online resources worldwide. Importantly, no portals or sites are available that can recognize which posted advertisements belong to real companies and which are fraudulent. This article aims to build a smart secured framework for detecting such issues using prediction models that can help in identifying the fraudulent jobs posted by fake companies using machine learning techniques. Moreover, this study used several ensemble machine learning (ML) techniques and yielded reliable outcomes for identifying and raising awareness about fraudulent job postings. Ultimately, this will save candidates time, effort, and money that they can devote to applying for real jobs while preserving the privacy and confidentiality of their information.

The rest of this study is structured as follows: the related work section discusses some of the existing solutions and approaches that are implemented in online recruitment fraud (ORF) followed by the step-by-step methodology including framework design, data collection, preprocessing, prediction models, and model evaluation. The later section discusses the results and analysis, while the last section concludes this study.

## Related Work

This section discusses the methods and techniques used for preventing fraudulent jobs as presented in previous studies. Several databases and other online resources were browsed for literature on related topics in order to better understand the techniques and data analysis used. The study of Anita et al. (2021) used logistic regression (LR), k-nearest neighbor (k-NN), Random Forest (RF), and deep learning (DL) algorithms for detecting fraudulent jobs from a large pool of real data and found that DL performed best. Another study by Tabassum et al. (2021) applied seven different ML algorithms and found the highest accuracy of two classifiers at 95.17%. The study of Alghamdi & Alharby (2019) created a prediction model using an RF algorithm for preventing fraudulent jobs and achieved 97.41% accuracy. Similarly, the study by Habiba, Islam & Tasnim (2021) built seven different models in which a deep neural network (DNN) outperformed others and achieved 98% accuracy in predicting fake job posts. The ensemble method approach utilized by AsmithaShree et al. (2021) involved training two single and one ensemble method and found the highest accuracy of the ensemble method for detecting fake enrollment. Another study by Mehboob & Malik (2021) applied the Xtreme Gradient Boost (XGB) algorithm to selected features of the same dataset and obtained 97.94% accuracy.

A report submitted by Ghosh et al. (2021) developed prediction models by training several classifiers for detecting online recruitment fraud and concluded that voting was the most accurate model, with an accuracy of 95.34%. The study of Mahbub & Pardede (2018) proposed a novel approach of adding contextual features to increase the accuracy of the detection model for identifying online recruitment fraud. A study conducted by Zuhair, Selmat & Salleh (2015) used several features of selection techniques for building reliable models based on the subset of features and concluded that the accuracy of the phishing detection model was highest among the examined models. Similarly, the study of Al-Garadi, Varathan & Ravana (2016) attempted to detect cyberbullying on Twitter and therefore trained several ML models based on the subset of features and found that RF achieved the highest results of 93% of F-measure.

A framework for detecting online recruitment fraud presented by Lal et al. (2019) used ensemble methods and achieved 95.4% accuracy on the same dataset, but the class imbalance issue was not resolved. A hierarchical clusters-based deep neural network (HC-DNN) was used for detecting fraud job placement by Kim, Kim & Kim (2019), who concluded that the proposed method outperformed other traditional methods. The study of Dutta & Bandyopadhyay (2020) used single and ensemble classifiers for identifying fraudulent jobs, and the ensemble methods performed well. Table 1 summarizes the related work.

**Table 1:** Summary of related work.

Ref.	Dataset	Dataset size	Methods	Preprocessing method(s)	Outperformed method(s)	Model’s accuracy
Anita et al. (2021)	Kaggle	18,000 records	LR, k-NN, RF, DL	Missing values removed	DL	98%
Tabassum et al. (2021)	Private	4,000 records	LR, AB, DT, RF Voting, LightGBM, GBoosting	Data cleaning, normalization, label encoder	LightGBM, GBoosting	95.17%
Alghamdi & Alharby (2019)	Kaggle	17,880 records	RF	Filled missing values in MS Excel, feature selection	RF	97.41%
Habiba, Islam & Tasnim (2021)	Kaggle	18,000 records	KNN, DT, SVM, NB, RF, DNN	Feature selection, conversion to categorical form	DNN	98%
Mehboob & Malik (2021)	Kaggle	17,880 records	NB, k-NN, DT, MLP, SVM, RF, XGB	Feature selection	XGB	97.94%
Ghosh et al. (2021)	Private	4,000	LR, AB, DT, RF, Voting, LightGBM, GBoosting	Data cleaning, feature scaling, normalization	Voting	95.34%
Mahbub & Pardede (2018)	Kaggle	17,880 records	DT, JRip, NB	Addition of contextual features	JRip rule-based	96.19%
Al-Garadi, Varathan & Ravana (2016)	Private	2.5 million tweets	RF	Oversampling	RF	AUC94.3%, F-score 93.6%
Lal et al. (2019)	Kaggle	17,880 records	ORFDetector	Feature extraction	ORFDetector	95.4%
Kim, Kim & Kim (2019)	Private	19,505 records	HC-DNN	Oversampling, PCA	HC-DNN	98.04%
Dutta & Bandyopadhyay (2020)	Kaggle	17,880 records	NB, k-NN, DT, RF, AB, GBoosting	Data cleaning, missing values removed	RF	98.27%

**Notes.**

LR, logistic regression; k-NN, k-nearest neighbor; RF, random forest; NB, naïve Bayes; DL, deep learning; AB, AdaBoost; DNN, deep neural network; MLP, multilayer perceptron; SVM, support vector machine; XGB, XGBoost; HC-DNN, hierarchical clusters-based deep neural network.

### Designing a framework

**Figure 1:**
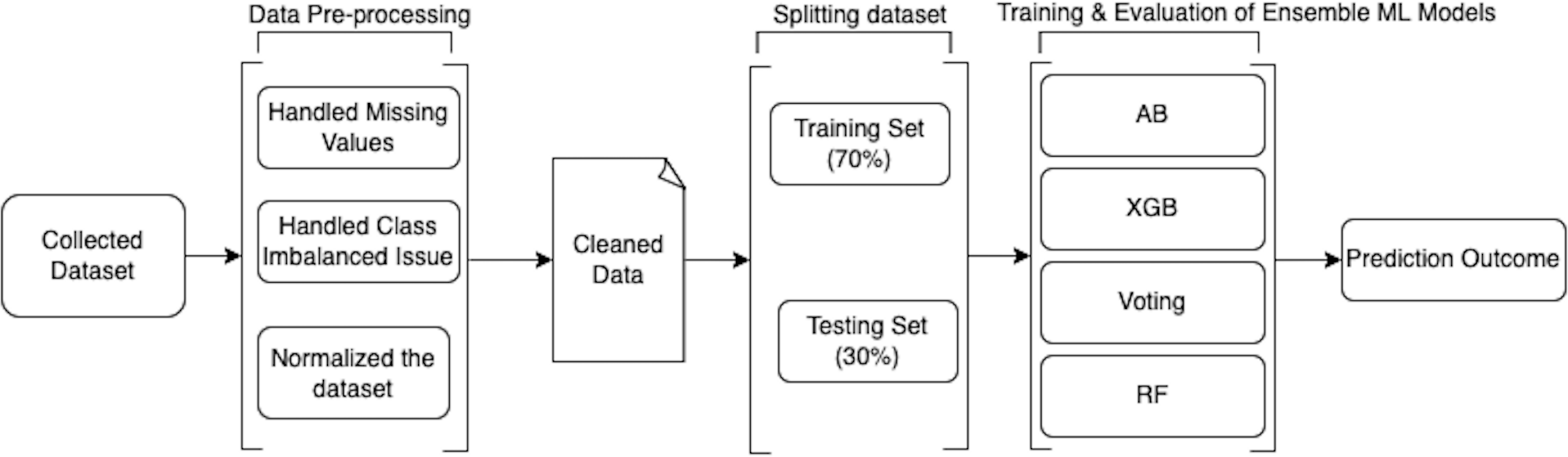
Design of the proposed work.

The methodology used to conduct this study and design a smart secured framework for detecting and preventing ORF using ensemble ML techniques is discussed in detail in the following sub-sections. Figure 1 shows the design of the proposed framework. Moreover, the whole implementation for data cleaning, analysis, and building prediction models was performed using Python 3.9 (https://www.python.org/downloads/release/python-390/).

## Data collection

**Figure 2:**
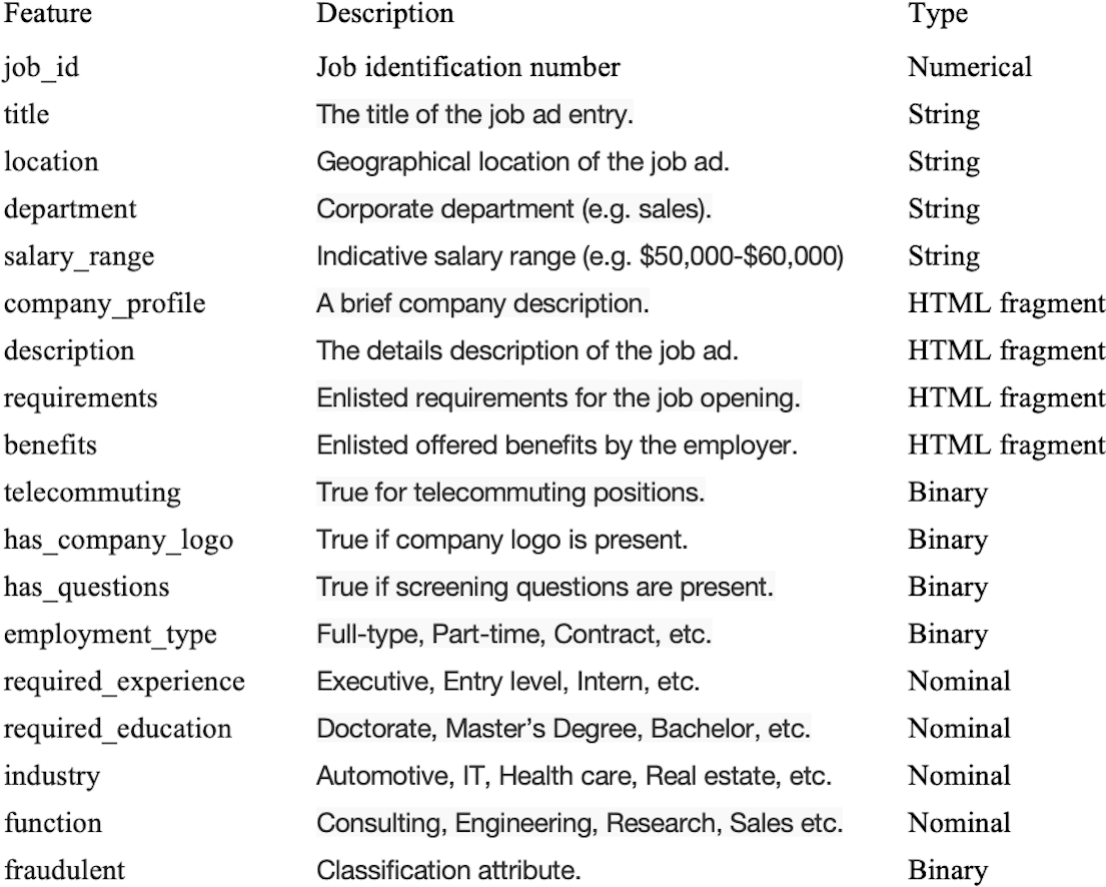
Dataset descriptions.

The first step in designing a smart secured framework was the requirement of data collection. Hence, the dataset was collected from a publicly available resource, Kaggle (Kaggle, 2021), originally harvested from the publicly available source of the University of the Aegean (Vidros et al., 2017). The dataset contains a total of 17,880 records, of which 866 records represent fraudulent jobs while the rest are real jobs. Moreover, the dataset contains a total of 18 features, including strings (four), HTML formats (four), binary features (five), numerical features (one), and nominal features (four). The target variable is a binary variable that shows whether or not the job is fraudulent. Figure 2 shows the dataset description and the types of its features.

### Data preprocessing

In predictive analytics, decisions are always based on the historical data from which the hidden patterns are extracted, and, based on the results, predictions are made for the unseen scenario (Ullah & Jamjoom, 2022a). Therefore, the data must be complete, clean, and reliable before the training of a model (Al-Mudimigh & Ullah, 2011; Al-Sudairi, Al-Mudimigh & Ullah, 2011).

**Figure 3:**
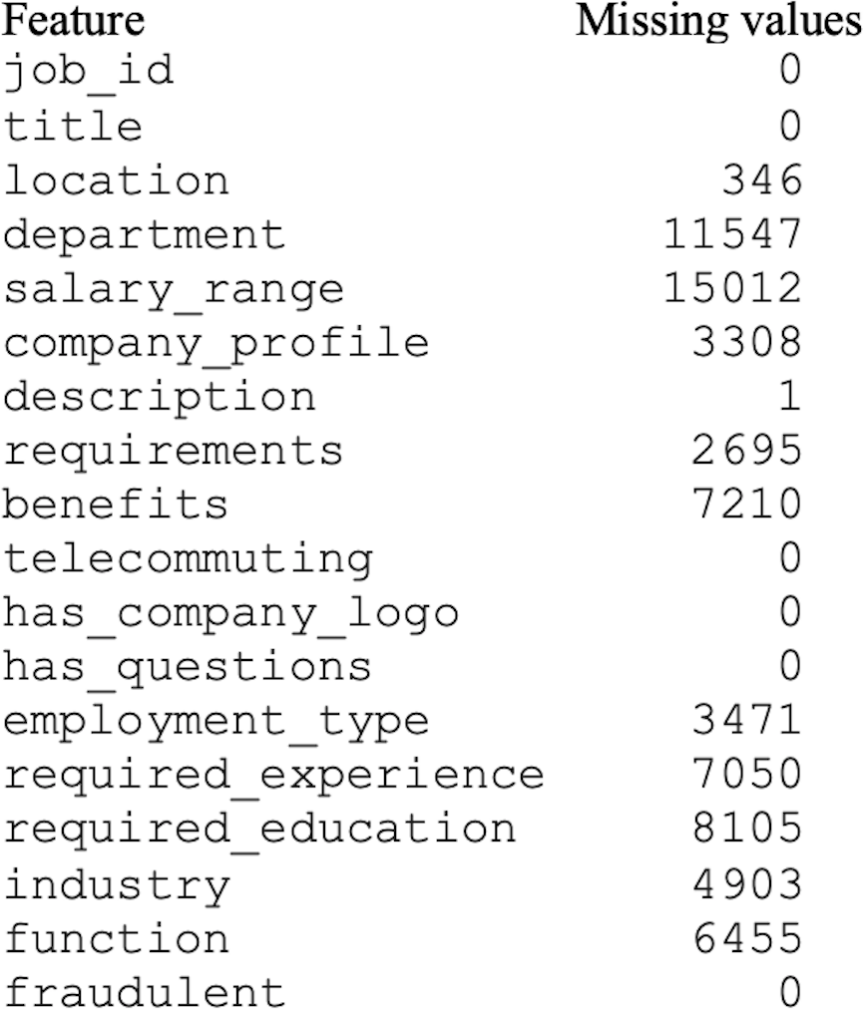
Dataset with missing values.

The original dataset consisted of numerous missing values, as shown in Fig. 3. The missing or null values could guide the classifier toward the wrong prediction (Hasan et al., 2020). In this study, the missing values were handled using the mode method. In the mode method, the most frequently occurring value is used to fill in the missing values.

**Figure 4:**
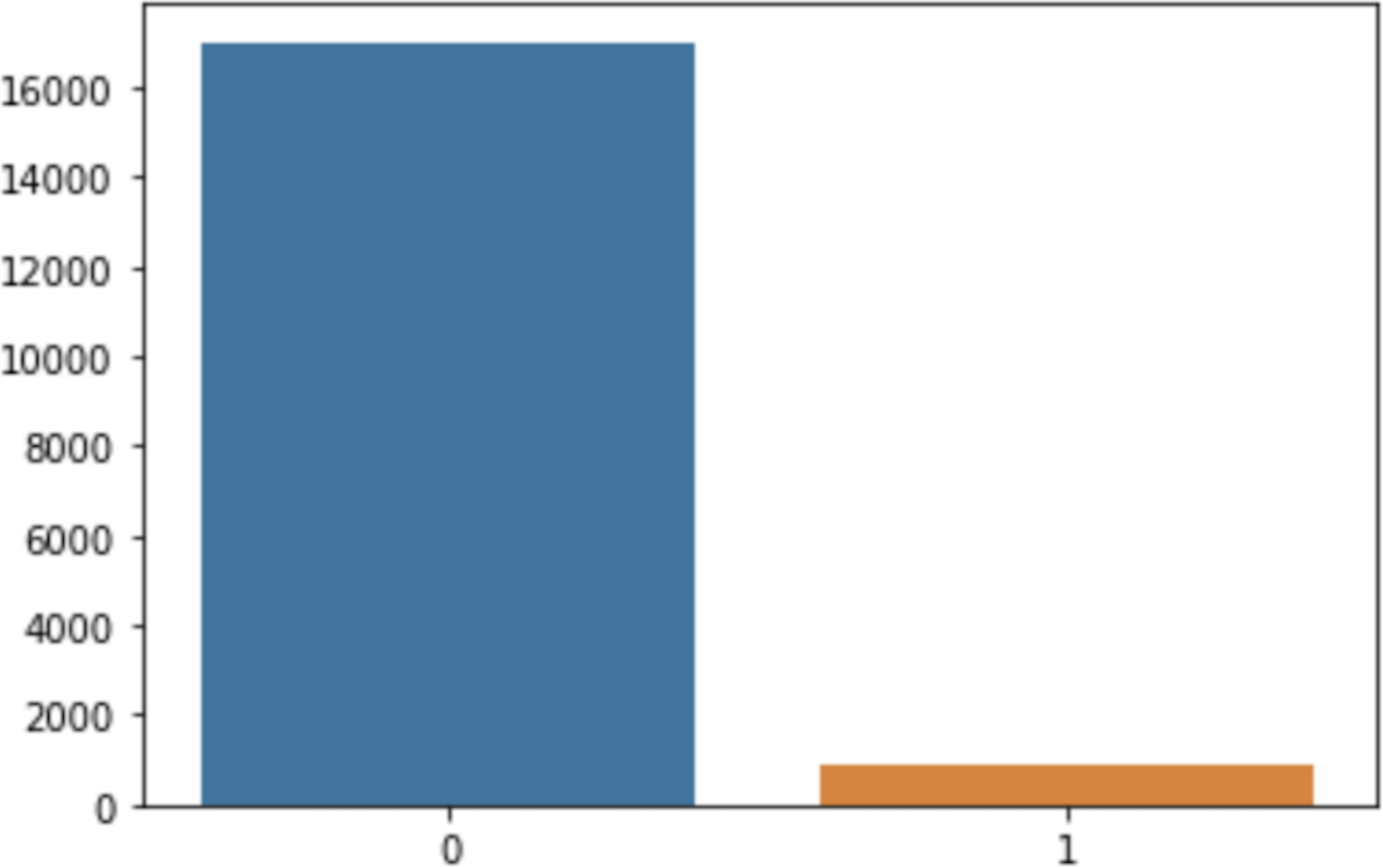
Imbalance classes.

Similarly, the dataset was hugely imbalanced, in that a class labeled 0 had 17,014 records while a class labeled 1 had only 866 records, as shown in Fig. 4. This class imbalance is a scenario in which the records of one class (typically a class of interest) are much less numerous than the records available for another class (Guzmán-Ponce et al., 2021; Ullah & Jamjoom, 2022b). In ML, data mining, and knowledge discovery, the class imbalance is considered challenging due to the biased favoritism of standard predictive models towards the majority class, because the likelihood of the actual values is presumed to be noise or the records are assigned to the majority class irrespective of the value of their attributes (García et al., 2020; Guzmán-Ponce et al., 2021), thus sacrificing the accuracy of the minority class (Elreedy & Atiya, 2019). The class imbalance problem can be resolved to generate extra data from the minority class and recover the shortage of data (Elreedy & Atiya, 2019). This was necessary to balance both classes for accurate model building. In this case, the class imbalance issue was handled using the oversampling method, in which both classes have an equal number of records. Likewise, the categorical data were transformed using Label Encoder. Finally, feature scaling was used to normalize the independent features.

### Prediction models

This study employed ensemble techniques for building prediction models. Unlike traditional ML techniques, ensemble methods utilize several algorithms together and integrate them in such a manner as to increase the prediction capability of the model and provide a single optimum solution to a problem (Hooda et al., 2021; Anifowose, 2021). The ensemble methods used for conducting this study are discussed in the following sub-sections.

#### AdaBoost (AB)

AdaBoost is an ensemble learning method that combines several algorithms to enhance the predictive ability of a model. This method utilizes decision tree (DT) as a base model in which each tree is trained to reduce the weakness of the previous DT by focusing on the misclassified data in the tree being trained that are boosted using weights (Mehta & Patnaik, 2021). This is an iterative method in which weights are utilized to train the data in each iteration until it confirms the accurate prediction of the misclassified data (Ullah et al., 2021).

#### XGBoost

XGBoost (eXtreme Gradient Boosting) is an ensemble technique that deploys a gradient boosted (GB) tree algorithm and is used to predict an output by combining multiple weaker or lower performance models (Mehta & Patnaik, 2021). This method trains multiple models in a steady and consecutive manner. GB is similar to AB, as both methods decide about the weaknesses of trained DTs; however, AB differentiates the weakness using weighted data, while GB uses gradients inside the loss function and the loss function shows the smartness of the model’s coefficients in fitting the unseen information (Kaliyar, Goswami & Narang, 2019).

#### Voting

Voting is an ensemble method that combines multiple classifiers and collects the output of each classifier, and the final prediction decision of a class is based on the maximum number of votes cast (Erdoğan & Namlı, 2019). Each prediction performed by the models is considered a vote, which is regarded as a measure of its accuracy. The more the number of votes cast for a class is decided for the final prediction (Cai et al., 2020).

In this study, the voting model combined four different models for predicting online advertised fake jobs—SVM, DT, Logistic Regression, and k-NN—and yielded better results.

#### Random Forest (RF)

RF is a widely used ensemble method that has several advantages: it is robust to noisy data, can resist overfitting and handling of missing values (Al-Abadi, 2018), has shown higher accuracy in several fields (Sarica, Cerasa & Quattrone, 2017), and has fewer classification errors (Ullah et al., 2021). This method accumulates multiple trees into a single ensemble forest and trains each tree using a bootstrap sample of the training set and independently sampled random subset of features (Al-Abadi, 2018). In RF, the central parameter for the classifier is the number of trees (Seker & Ocak, 2019). In this study, the number of trees is set to 10 for building the RF model.

### Model evaluation

The model evaluation is a process of assessing the final trained models’ predictions and comparing those predictions against the actual data, which is commonly known as test data (Smith & Frank, 2016). This can be done using several methods, such as by using the training set, supplied test set, cross-validation, or percentage split (Smith & Frank, 2016; Ahmad et al., 2021). Moreover, for model evaluation, using the whole dataset for training and testing can lead to the risk of overestimation of the values of a model, because the same data have been seen by the model during training (Smith & Frank, 2016). This method can be useful if someone is interested only in a descriptive model and not a predictive one (Brownlee, 2019; Ksibi et al., 2022). However, this method is generally not recommended (Mitchell, 1997; Al-Mudimigh, Ullah & Alsubaie, 2011; Smith & Frank, 2016; Brownlee, 2019). It is a real ML challenge to predict unseen data based on the hidden patterns of historical data that have not been seen during training (Smith & Frank, 2016; Ullah & Jamjoom, 2022c).

**Figure 5:**
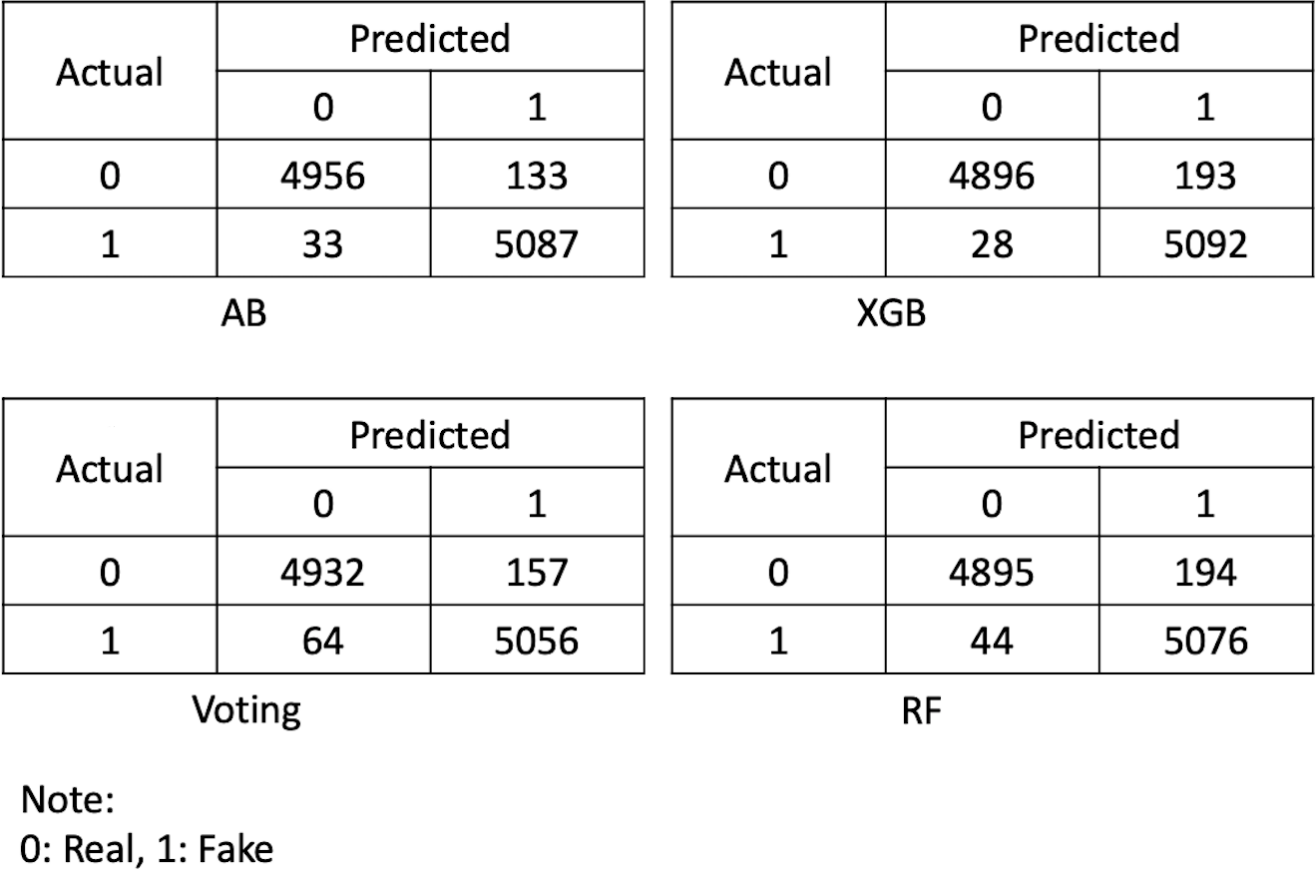
Confusion matrix for the ensemble models.

In this study, the percentage split method was used to separate the training and testing set. Therefore, the dataset was divided into two sets, with 70% of the data used for training the prediction models and the remaining 30% of the data being used for testing purposes. Thus, all the parameters of each classifier were set during the training phase, and a confusion matrix for each ensemble model was achieved, which further explains the four important measures of true positive, true negative, false positive, and false negative. These are the fundamental measures that are used for evaluating the model performance in terms of precision, recall, F-measure, and receiver operating curve (ROC). Every evaluation measure is computed using its equation, as shown below. Figure 5 shows the confusion matrix of all ensemble methods used for identifying the online advertised fraudulent job.

(1) \begin{eqnarray*}Accuracy& = \frac{TP+TN}{TP+TN+FP+FN} \end{eqnarray*}

(2) \begin{eqnarray*}Precision& = \frac{TP}{TP+FP} \end{eqnarray*}

(3) \begin{eqnarray*}RecallorSensitivity& = \frac{TP}{TP+FN} \end{eqnarray*}

(4) \begin{eqnarray*}\mathrm{F}-measure& = \frac{(2\ast Precision\ast Recall)}{Precision+Recall} \end{eqnarray*}

## Results and Discussion

As discussed above, the dataset was divided into two sets, with 70% used for training the models and the remaining 30% used to assess the models’ performance against the predicted outcomes. The entire implementation of the training and testing phases of the prediction models and other analyses was performed using Python 3.9. Moreover, this study attempted to implement other ensemble methods such as bagging and stacking; however, the results achieved were not significantly different from those of the existing work. As a result, the methods selected for conducting this study were based on the accomplishment of higher accuracy rates and the development of best-fit models. Based on (1), the accuracies of the trained ensemble models are shown in Table 2.

**Table 2:** Accuracies and kappa values of the ensemble models.

Classifier	Accuracy (%)	Kappa Value
AdaBoost (AB)	98.374	0.9675
XGBoost (XGB)	97.835	0.9567
Voting	97.835	0.9567
Random Forest (RF)	97.669	0.9534

As shown in Table 2, AB performed best in terms of accuracy at 98.374%, followed by XGB and voting with identical accuracies of 97.835%, and RF with an accuracy of 97.669%. The trained ensembled model performed well in terms of accuracy, which shows the model is reliable for detecting and preventing the online advertisement of fake jobs.

The Kappa value (Cohen, 1960) is a measure that equates observed accuracy with predicted accuracy. This is an important technique when two or more independent methods are investigating the same problem (Abbas et al., 2018). The kappa value has different thresholds in ranges; however, a value larger than 0.75 is excellent (McHugh, 2012). In Table 2, the kappa values for all ensemble methods are higher than the excellent threshold, contributing to the significance and reliability of trained models for predicting and preventing ORF and online advertisement job scams.

Similarly, the accuracies of the ensemble methods used for predicting fake job postings were also measured using precision, sensitivity, and F-measures. Precision is defined as the fraction of accurately predicted positive data to all data that is predicted to be positive (Powers, 2020). Precision, also known as the positive predictive value or confidence of a model (Lalkhen & McCluskey, 2008), is calculated as per equation (2). Sensitivity, which is also referred to as recall, is the fraction of accurately predicted positive data to all data in an actual class (Powers, 2020). Sensitivity is calculated using equation (3). The weighted mean of precision and sensitivity is referred to as F-measure (Van Rijsbergen, 1979). Table 3 shows the precision, sensitivity, and F-measure of the ensembled methods used for predicting fraudulent online job advertisements.

According to Table 3, the values of precision, recall, and F-measures are higher for all ensemble methods in which precision is higher at 99%, F-measure is 98%, and sensitivity is varied in that AB and voting have 97% but XGB and RF has 96%. The overall performance of the ensemble methods used for predicting fraudulent job postings shows the reliability of the models to be used as a decision support system for preventing ORF.

Furthermore, the ensemble models were also evaluated using the ROC curve, which is the representation of the true positive rate and false positive rate in a graphical form with different thresholds that demonstrate the analytical ability of a binary classifier (Kumar & Indrayan, 2011). The ROC curve analyzes the precision and recall in a more sophisticated manner, in that high precision shows a low false positive rate and high recall shows a low false negative rate, as evident in the accurate and positive outcomes of a classifier (Abbas et al., 2018). Therefore, the ROC curve is more advantageous than single precision and recall. Additionally, in the ROC curve, a classifier has the highest accuracy when the curve is closest to the upper left corner (Kumar & Indrayan, 2011).

**Figure 6:**
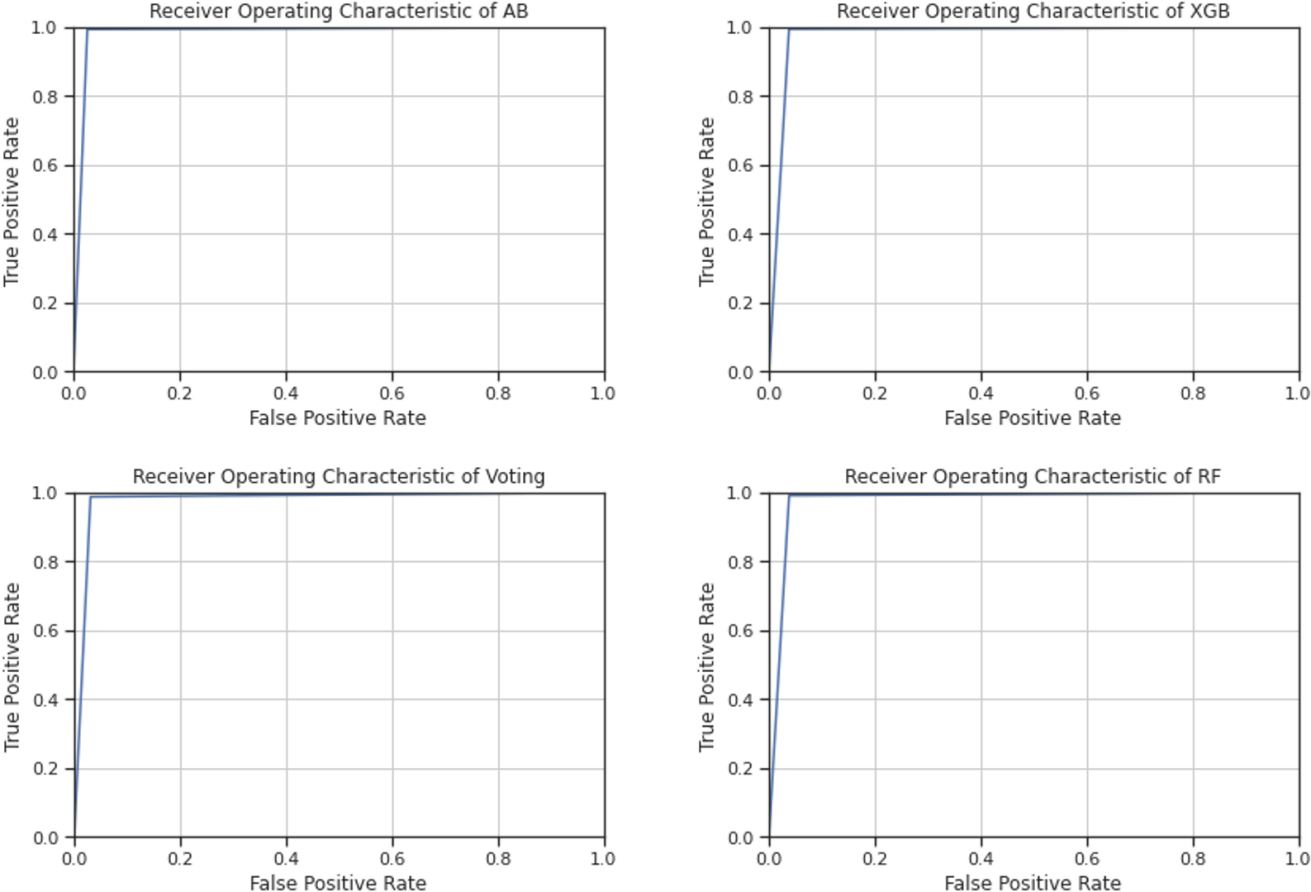
ROC curves of the ensemble methods.

Figure 6 shows the ROC curves of all ensemble methods. As can be seen in Fig. 6, the curve is very close to the upper left corner in all ensemble methods, showing the reliability of the models for use in predicting online recruitment fraud.

**Table 3:** Evaluation measures of the ensemble methods.

Classifier	Precision	Recall	F1-Measure
AdaBoost	0.99	0.97	0.98
XGBoost	0.99	0.96	0.98
Voting	0.99	0.97	0.98
Random Forest	0.99	0.96	0.98

Moreover, the results achieved by the proposed models for detecting ORF have been compared with the existing studies. As AB outperformed other models, therefore, we used to compare the results of AB with existing works. Table 4 demonstrates the comparison of the proposed AB with existing models.

As shown in Table 4, the proposed AB method outperformed other existing methods. In Mehboob & Malik (2021) the same dataset was used for training different ML models to detect ORF. In the pre-processing steps, duplicates and blank records were deleted from the dataset. The class imbalance was handled. The authors used a two-step method to finalize a set of best-fit features in that firstly they combined additional features with the existing features. Then, feature selection was applied to choose top ranked feature using statistical methods. The highly correlated features were selected, and low-ranked features were dropped, thus finalized with a dataset containing 18 features. Secondly, the top 18 features of the first step were reconsidered to finalize the best combination of features. In the second step, the wrapper method was utilized for achieving an optimal combination of features. Finally, they came up with 13 features that were used to train seven different ML models. As a result, XGB outperformed others with an accuracy rate of 97.94%.

Similarly, Dutta & Bandyopadhyay (2020) used the same dataset for training the proposed models. Prior to implementation, the dataset was pre-processed in that the missing values, nonrelevant features, and spaces were removed from the dataset. A multi-step practice was utilized to balance the dataset. The processed dataset was used to train several ML models in which RF outperformed other models.

**Table 4:** Comparison of the proposed AB model with existing models.

Ref.	Dataset	Dataset size	Method	Preprocessing method(s)	Model’s accuracy
Mehboob & Malik (2021)	Kaggle	17,880 records	XGB	Feature selection	97.94%
Ghosh et al. (2021)	Private	4,000 records	Voting	Data cleaning, feature scaling, normalization	95.34%
Dutta & Bandyopadhyay (2020)	Kaggle	17,880 records	RF	Data cleaning, missing values removed	98.27%
Alghamdi & Alharby (2019)	Kaggle	17,880 records	RF	Fill in missing values in MS Excel, feature selection	97.41%
Proposed	Kaggle	17,880 records	AB	Fill in missing values, oversampling, label encoder, normalization	98.37%

A study conducted by Alghamdi & Alharby (2019) used a dataset similar to the previous two studies for detecting fraud in ORF. The dataset was pre-processed and feature selection using Weka built-in filters was applied. The processed dataset was utilized to train the RF model using the Weka tool and achieved the highest accuracy rate of 97.41%.

The results shown in the above tables and figures demonstrate that the ensemble models trained for predicting employment scams are reliable and can be part of a decision-making process to select real job offerings for employment. The ensemble models’ reliability and trustworthiness were analyzed using several evaluation methods, and each individual method provides significant outcomes. The overall performance of each ensemble method is high, with AB performing best in terms of overall accuracy, precision, sensitivity, F-measure, and ROC. Similarly, XGB, voting, and RF performed well; though their overall accuracies have some variations, they can nonetheless contribute to good decision-making in identifying ORF in advertised jobs.

## Conclusion

Four ensemble ML methods were applied to build a secured framework for detecting and preventing ORF and preserving the privacy of candidates applying for jobs online. Before building the framework, several preprocessing steps were taken to handle missing values, noise, and class imbalance problems. The proposed framework built based on cleaned data yields better outcomes, as AB performed best in terms of accuracy, precision, sensitivity, F-measure, and ROC curve. The AB method in the proposed framework outperformed compared to the existing methods in the literature demonstrating the reliability of the model to be used for detecting ORF. Similarly, the evaluation of XGB, voting, and RF achieved better performance in terms of accuracy and other measures. Hence, the methods used in the framework show significant contributions of the models for detecting ORF and preventing online job scams from fraudsters. Moreover, the overall performance of the ensemble methods used in the framework for predicting and preventing online recruitment scams shows the reliability of the models to be used as a smart decision-making process for solving the problem of employment scams.

##  Supplemental Information

10.7717/peerj-cs.1234/supp-1 Supplemental Information 1 Real or fake job postings

10.7717/peerj-cs.1234/supp-2 Supplemental Information 2 Code

## Additional Information and Declarations

### Data Availability

The following information was supplied regarding data availability: The raw data and code are available in the Supplemental Files.
